# NUP155 and NDC1 interaction in NSCLC: a promising target for tumor progression

**DOI:** 10.3389/fphar.2024.1514367

**Published:** 2024-12-10

**Authors:** Kai-Min Li, Li-Fei Meng, Zhi-Hao Yang, Wen-Tao Hu

**Affiliations:** Department of Thoracic Surgery, The First Affiliated Hospital of Ningbo University, Ningbo, China

**Keywords:** NUP155, NDC1, NSCLC, nucleoporins, nuclear pore complex

## Abstract

**Background:**

NUP155 was reported to involve breast invasive carcinoma and hepatocellular carcinoma. We hypothesized that NUP155 and NDC1impacted the progression of NSCLC.

**Methods:**

The dataset was analyzed to find differentially expressed genes. Functional enrichment analysis and Kaplan-Meier survival analysis were performed for differentially expressed genes. Western blot, Clone formation assay, Transwell assay and CCK-8 assay were performed to determine the performance and role of the target gene in NSCLC.

**Results:**

The research found that the NUP family played a role in various diseases. Differential expression analysis and survival analysis were performed to identify 6 related-genes, including NUP155, NDC1, KPNA2, MAD2L1, NUP62CL, and POM121L2NUP155 and NDC1 could interact with NUP53, respectively. This effect was necessary to complete the assembly of the nuclear pore complex.

**Conclusion:**

NUP155 interacted with NDC1 to complete the assembly of the nuclear pore complex, which promoted the development of NSCLC. Our study demonstrated that NUP155 was expected to be a potential target for the treatment of NSCLC.

## 1 Introduction

Cancer is a great threat to human health and life, and the treatment for cancer is an important topic in the medical field. The number of patients who die from lung cancer is reported to be a quarter of cancer-related deaths. Non-small cell lung cancer (NSCLC) is the subtypes of lung cancer, and its 60-month overall survival rate remains low (Srivastava et al., 2022; Duma et al., 2019). Although early screening has the opportunity to prolong the life of patients, it is more important for patients to have effective treatment. In recent years, the development of targeted therapies has improved clinical outcomes for some patients to some extent (Osmani et al., 2018). Therefore, it is particularly important to find targets to further prolong the survival of NSCLC patients. NUP155 is expected to be such a target.

The nuclear pore complex (NPC) is a unique transmembrane transporter complex that acts as an import and export channel for molecules inside and outside the nucleus (Davis, 1995; Sakuma and D'Angelo, 2017; Hurt and Beck, 2015; Reza et al., 2016). NPC deficiency has an impact on gene expression and growth, and has been linked to viral infections, neurodegenerative diseases and cancer. NUP155 is one of the nucleoporins, which affects the structural assembly of NPCs. Studies have shown that NUP155 is associated with the occurrence of a variety of cancers, and it has been found to have mutations in a variety of tumors, such as cervical adenocarcinoma, endometrial cancer, melanoma cancer (Savci-Heijink et al., 2016; Engqvist et al., 2020; Wang et al., 2024). In hepatocellular carcinoma, full p21 (CDKN1A) mRNA translation is required to regulate FTSJ1 transcription through the interaction of HDAC4 with Nup155 upon p53 activation. At the same time, Nup155 is inhibited by p53 in a p21-dependent manner (Holzer et al., 2019). However, the mechanism of NUP155 in NSCLC remains currently unclear.

NDC1 is a transmembrane nuclear porin that, together with another nuclear porin, plays a role in securing large NPCs in the nuclear envelope (Bindra and Mishra, 2021). Altered NDC1 expression has been found in a variety of cancers. Downregulation of NDC1 inhibits DNA replication and cell cycle-related genes, such as PCNA and CYCLINB1, thereby reducing cell proliferation and migration. In mice, inhibition of NDC1 leads to a decrease in cell migration and tumorigenicity, inducing apoptosis (Qiao et al., 2016). NDC1 has been reported to play a regulatory role in esophageal squamous cell carcinoma (ESCC) and cervical cancer (CC) (Bindra and Mishra, 2021).

NUP155 and NDC1 are both nucleoporin members. NDC1 involved in nuclear pore complex assembly and nuclear pore localization. The channel nucleus pores of the Nup62/58/54 heterotrimers are anchored to the central support. 6 Nup155 molecules interact with the central scaffold and, together with NDC1-ALADIN heterodimers, immobilize the IR subunit on the nuclear membrane and outer ring (Huang et al., 2022). However, the role of NUP155 and NDC1 in NSCLC remain unknown. In this paper, The preliminary role of NUP155 and NDC1 was confirmed in NSCLC, providing a basis for follow-up studies and two therapeutic target for NSCLC patients.

## 2 Materials and methods

### 2.1 Data sources

NSCLC dataset was downloaded from the Cancer Genome Atlas Program (TCGA) database (https://www.cancer.gov/ccg/research/genome-sequencing/tcga). The cleansing of the initial data is performed using Perl programming to remove any duplicate or incomplete observations from the dataset, such as no survival information and age 0 samples. The next step was to normalize and annotate the data to meet the requirements of subsequent analysis. The valid data contained 446 NSCLC samples and 80 normal samples. These data were analyzed to find differentially expressed genes. NUP family related-gene set was download from database (https://www.genecards.org/).

### 2.2 Analysis of differential expression genes

Three commonly used algorithms, DESeq2, EdgeR and Limma, were utilized to analyze the differentially expressed genes in the datasets, and each algorithm obtained the high-expression and low-expression gene sets, respectively. Subsequently, the high-expression gene set and the low-expression gene set were intersected, and finally the differentially expressed genes were obtained. (|log FC (fold change) | ≥1.0 and P value < 0 .05).

### 2.3 Functional enrichment analysis

Gene Ontology (GO) and Kyoto Encyclopedia of Genes and Genomes (KEGG) function enrichment analysis were performed on differentially expressed gene sets. Among them, GO annotation was divided into three categories, namely Biological Process (BP), Cellular Components (CC) and Molecular Function (MF), through which the functions of genes could be described in various aspects. KEGG was used to analyze the gene pathway. Finally, GSEA enrichment analysis was performed on the differentially expressed gene sets with GO and KEGG as annotations, respectively.

### 2.4 Kaplan-Meier survival analysis

Kaplan-Meier survival analysis was performed for 21 differentially expressed genes, including NDC1, NUP155, HSF2BP, SASS6, TPRG1, KPNA2, MAD2L1, MAPK10, NUP62CL, NUP62CL.1, POM121L2, POM121L2.1, NUP210L, AURKA, NUP210, IPO4, NXF3, ZFP36, RANBP3L, XPO5, TUBG1. NUP62CL.1 and POM121L2.1 were duplicated with NUP62CL and POM121L2. Therefore, the survival rate of 19 genes in patients was estimated, the survival curve was drawn.

### 2.5 Western blot

Total protein was extracted from the tissues and their content was determined, followed by SDS-PAGE polyacrylamide gel electrophoresis, in which the eluted gel was immersed in transfer buffer for 15 min and the membrane was stained with 1 × ponceau stain for 5 min. The membrane was washed with 10 mL of 1 × TBST for 5 min at room temperature and was washed three times. 5 mL of skimmed milk powder blocking solution was incubated at room temperature for 2 h 5 mL of primary antibody dilution buffer was added for 2 h at room temperature. The primary antibody was recycled. Primary antibody information was listed as follow: NUP155 (1:1,000, Cat No. 66359-1-Ig, Proteintech, United States), NDC1 (1:1,000, Cat No. A17727, Abclonal, United States), β-actin (1:1,000, Cat No. 66009-1-Ig, Proteintech, United States), Vimentin (1:1,000, Cat No. 60330-1-Ig, Proteintech, United States) and E-cadherin (1:1,000, Cat No. 20874-1-AP, Proteintech, United States). The secondary antibody (1:10,000, Abbkine, China) was added and was shaken gently for 1 h at room temperature. The membrane was washed with 10 mL of TBST 3 times for 5 min each time. The target protein on the membrane was developed by chemiluminescence and the results were analyzed.

### 2.6 Clone formation experiment

Clone formation experiment was carried out to observe the colony formation of cells and was detected the proliferation ability of cells. The pretreated NSCLC cells were digested with trypsin and collected, then resuspended into single-cell suspensions using complete medium. The cell suspension concentration was adjusted to 1,000 cells/mL and cells were seeded into 6-well plates of 1,000 cells per well. After 15 days of incubation, the number of clones was observed, during which the fluid was fed every 3 days and the cell status was observed. The cells were then washed with PBS (Gibco, United States), and 1 mL of 4% paraformaldehyde (Servicebio, China) was added to each well to fix the cells for 40 min, and after washing again, 1 mL of crystal violet staining solution (Beyotime, China) was added to each well for staining for 20 min. The number of clones formed was counted under the microscope and each hole and the entire 6-well plate were photographed. Cell images were processed using ImageJ software.

### 2.7 Transwell experiment

50 μL of serum-free culture medium was added to each well of the Transwell chamber (Corning, United States) and the basement membrane was hydrated at 37°C for 30 min. Cells were starved for 24 h. After digesting the cells, centrifugation was performed and the culture was discarded. The cells were washed with 2 times using PBS and were resuspend in serum-free medium containing BSA. The cell density was adjusted to 5 × 10^5^/mL. 100 μL of the cell suspension was added to the Transwell chamber. Cells were cultured at 37°C, 5% CO_2_, 90% humidity for 24 h. The chamber was removed, and the culture was discarded. 600 μL of 4% paraformaldehyde (Servicebio, China) fixative solution was added. 600 μL of crystal violet staining solution (Beyotime, China) was added after discarding the fixative solution. The chamber was placed for staining for 10 min. After air-drying, the migration of the cells was observed and photographed under a microscope (Olympus Fluoview, Tokyo, Japan).

### 2.8 CCK-8 experiment

100 μL of cell suspension was added to a 96-well plate and was placed at 37°C for pre-incubation. 10 μL of the solution was added to be tested at different concentrations such as 300, 100, 33.3, and 11.1 μM to each well, and the solution was incubated again at 37°C for 24 h. The CCK-8 solution was thawed at room temperature and wa s centrifuged. 10 μL of CCK-8 solution was added to each well and was incubated at 37°C for 3 h. The optical Density was determined at 450 nm with a microplate reader. The results were processed and analyzed using Excel and Graphpad Prism.

### 2.9 Scratch wound healing assay

The marker lines were drawn on the bottom surface of the six-well plate. Cells were seeded into six-well plates of 5 × 10^5^ per well. A 200 μL tip which was perpendicular to the plate and marker lines was used to make cell scratches. And then, pictures were taken with a microscope as a 0 h control. The old medium was discarded and wash three times with sterile PBS to rinse off the scratched cells. Fresh serum-free medium was added. The width of the scratch was observed with a microscope and photos were taken. ImageJ software was used for analysis. The above experiments were repeated for cancer cells with NUP155 knockdown and NUP155 overexpression, and photographed and analyzed.

### 2.10 Statistics

Statistical analysis was performed using GraphPad Prism version 10, GraphPad Software, and R statistical programming language (www.R-project.org). Survival analysis was estimated with the use of the Kaplan-Meier method and the log-rank test. Two-way analysis of variance (ANOVA) was performed followed by a Tukey *post hoc* test. The T test was used for comparison between groups. Statistical significance was defined as p* < 0.05, p** < 0.01, p** < 0.001 and was determined by 2-tailed Student’s t-test. All experiments were performed at least three times.

## 3 Results

### 3.1 Differentially expressed genes were analyzed

**FIGURE 1:**
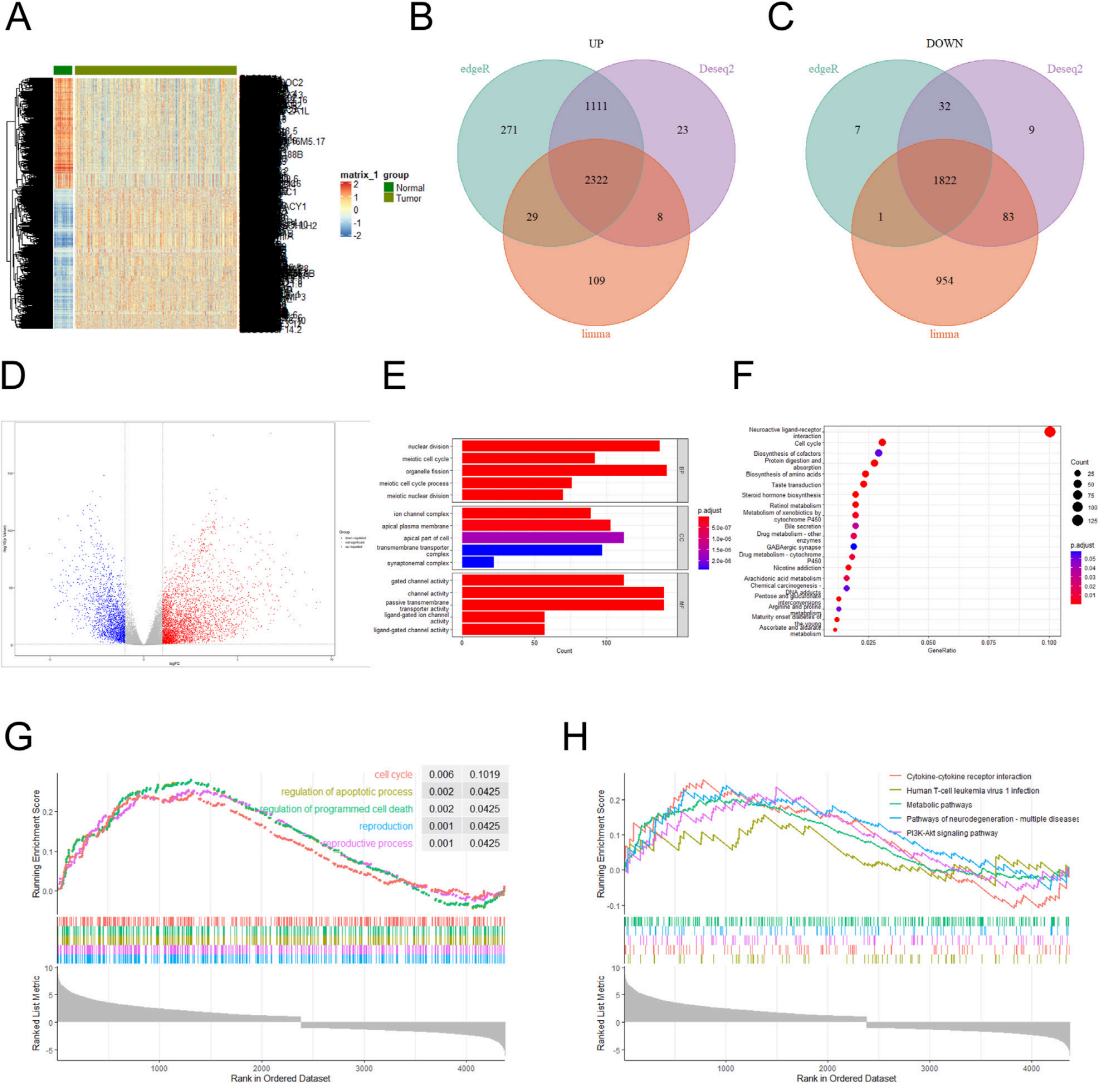
Differentially expressed genes in the NSCLC dataset were analyzed. **(A)** Differentially expressed genes in the NSCLC dataset were analyzed. **(B)** Highly expressed genes were analyzed using DESeq2, EdgeR, and Limma packages, respectively. A total of 3,464 high-expression genes were identified in the DESeq2 package, 3,733 high-expression genes in the EdgeR package, and 2,468 high-expression genes in the Limma package. Three sets of high-expression genes were intersected, and 2,322 high-expression genes were obtained. **(C)** Lowly expressed genes using DESeq2, EdgeR, and Limma packages were analyzed, respectively. A total of 1,946 low-expression genes were identified in the DESeq2 package, 1,862 low-expression genes in the EdgeR package, and 2,860 low-expression genes in the Limma package. Three sets of low-expression genes were intersected, and 1,822 low-expression genes were obtained. **(D)** Volcano plots were used to show the magnitude of changes in differentially expressed genes. **(E)** GO functional enrichment analysis was performed on the differentially expressed genes. **(F)** KEGG pathway enrichment analysis was conducted for the differentially expressed genes. **(G)** GSEA enrichment analysis was performed on the differentially expressed genes, and the annotated information was GO. **(H)** GSEA enrichment analysis was performed on the differentially expressed genes, and the annotated information was KEGG.

The NSCLC dataset was downloaded from the TCGA database, and the aberrantly expressed genes in it were shown by heat map (Figure 1A). Three difference analysis tools, DESeq2, EdgeR and Limma packages, were used to analyze the high-expression and low-expression genes respectively, and at the intersection, 2,322 high-expression genes and 1,822 low-expression genes were obtained (Figures 1B, C), and the analysis results were presented by volcano map (|log FC (fold change) | ≥1.0 and P value < 0. 05) (Figure 1D). Performing a GO enrichment analysis on the set of the differentially expressed genes, we found that the differentially expressed genes were mainly enriched in categories such as nuclear division, channel activity, passive transmembrane transporter activity, organelle fission and gated channel activity (Figure 1E). The result of KEGG enrichment analysis indicated that the differentially expressed genes were mainly concentrated in Neuroactive ligand-receptor interaction and Cell cycle (Figure 1F). GSEA enrichment analysis of highly expressed genes was conducted with KEGG annotation information, and the results showed that the top five enrichment pathways were Cell cycle, regulation of apoptotic process, regulation of programmed cell death, reproduction, and reproductive process (Figure 1G). When the annotation information of lowly expressed genes was analyzed, the top five enrichment pathways were Cytokine-cytokine receptor interaction, Human T-cell leukemia virus 1infection, Metabolic pathways, Pathways of neurodegeneration-multiple diseases and PI3K-Akt signaling pathway (Figure 1H). The results of KEGG, GO and GSEA confirmed that Cell cycle and nuclear division was important feature of cancers (Nelson et al., 2024; Liu et al., 2024). NUP155 is one of the nucleoporins, which affects the structural assembly of NPCs (Savci-Heijink et al., 2016; Engqvist et al., 2020; Wang et al., 2024). Nucleoporins plays an important role in cell cycle and nuclear division of NSCLC cells (Jagot-Lacoussiere et al., 2015). However, the mechanism of NUP155 in NSCLC remains currently unclear.

### 3.2 NUP155 and NDC1 were identified as study subjects

NPCS were composed of NUPS (7). It had been reported in the literature that nucleoporin played a role in a variety of cells that affected development, for example, NUP188 was a candidate gene for congenital heart disease, and knocking out this gene in Xeno-pus embryonic development would lead to left-right patterning disruption, and genes such as NUP98 and NUP153 had been implicated in the import or integration of HIV-1 (Reza et al., 2016). Based on this, we tried to study the NUP family to find a therapeutic target for NSCLC.

**FIGURE 2:**
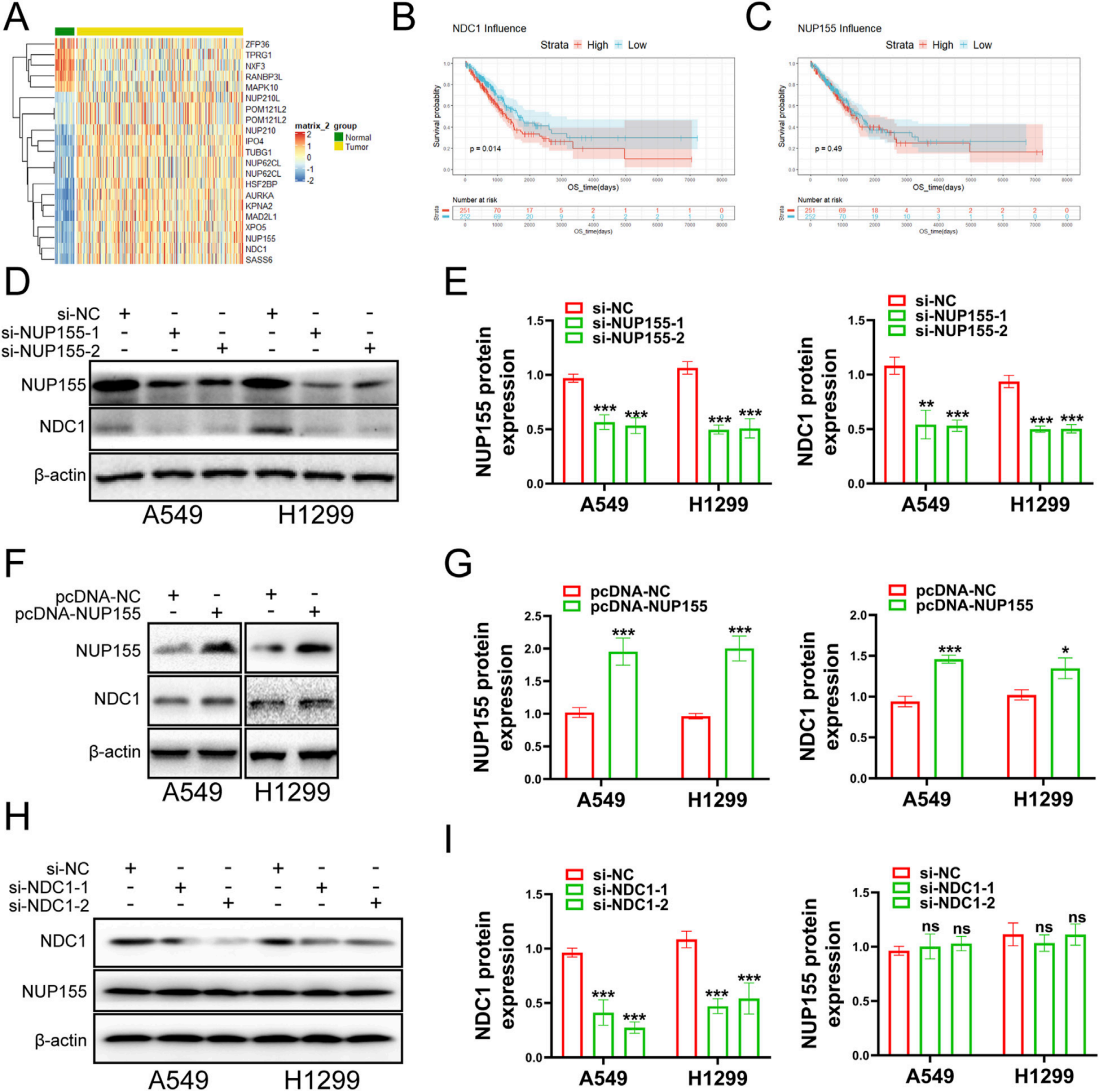
Examines the role of NUP155 in cancer cells. **(A)** The intersection of NUP family and differentially expressed genes yielded 21 differentially expressed genes. NUP62CL.1 and POM121L2.1 were duplicated with NUP62CL and POM121L2. **(B)** Survival analysis of NDC1. **(C)** Survival analysis of NUP155. **(D)** NUP155 was knocked down, and Western blot was used to detect the protein expression changes of NUP155 and NDC1. **(E)** Histogram was used to show the Western blot results of NUP155 and NDC1. **(F)** Protein expression changes of NUP155 and NDC1 in cancer cells were detected after overexpression of NUP155. **(G)** Histogram was utilized to display the Western blot results of NUP155 and NDC1. **(H)** After NDC1 knockdown, the protein expression changes of NDC1 and NUP155 in cancer cells were detected. **(I)** Histogram was used to show the results of NUP155 and NDC1.

**FIGURE 3:**
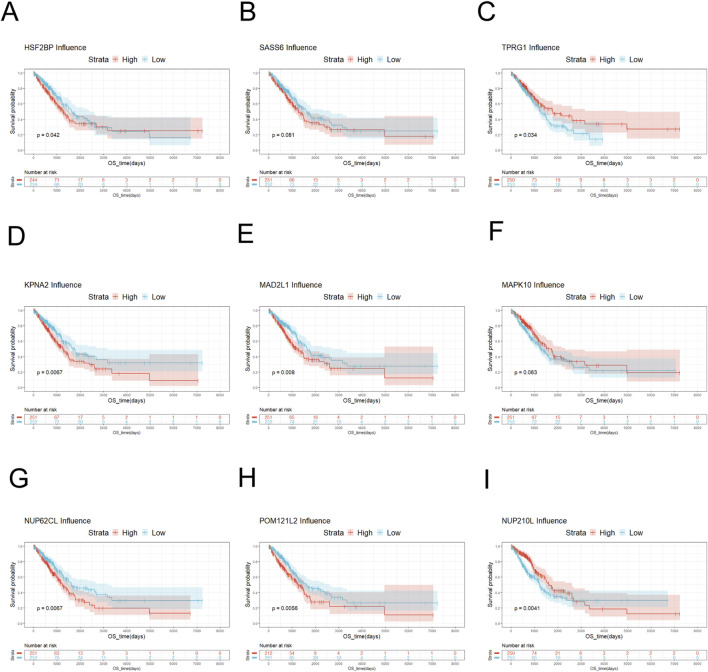
Survival analysis of key genes. **(A)** Survival analysis of HSF2BP showed no significant difference in survival probability between low-risk HSF2BP and high-risk HSF2BP. **(B)** Survival analysis of SASS6 exhibited that the survival probability of low-risk SASS6 was slightly higher than that of high-risk SASS6. **(C)** Survival analysis of TPRG1 displayed that the survival probability of high-risk TPRG1 was higher than that of low-risk TPRG1. **(D)** Survival analysis of KPNA2 manifested that low-risk KPNA2 had a higher survival probability than high-risk KPNA2. **(E)** Survival analysis of MAD2L1 demonstrated that low-risk MAD2L1 had a higher survival probability than high-risk MAD2L1. **(F)** Survival analysis of MAPK10 showed that the survival probability of high-risk MAPK10 was higher than that of low-risk MAPK10. **(G)** Survival analysis was performed on NUP62CL, and the result indicated that the survival probability of low-risk NUP62CL was higher than that of high-risk NUP62CL. **(H)** Survival analysis was performed on POM121L2, and the probability of survival was higher for low-risk POM121L2 than for high-risk POM121L2. **(I)** Survival analysis was performed on NUP210L. When the overall survival time was less than 3,700 days, the survival probability of high-risk NUP210L was higher than that of low-risk NUP210L. When the overall survival was greater than 3,700 days, the probability of survival was higher for low-risk NUP210L than for high-risk NUP210L.

**FIGURE 4:**
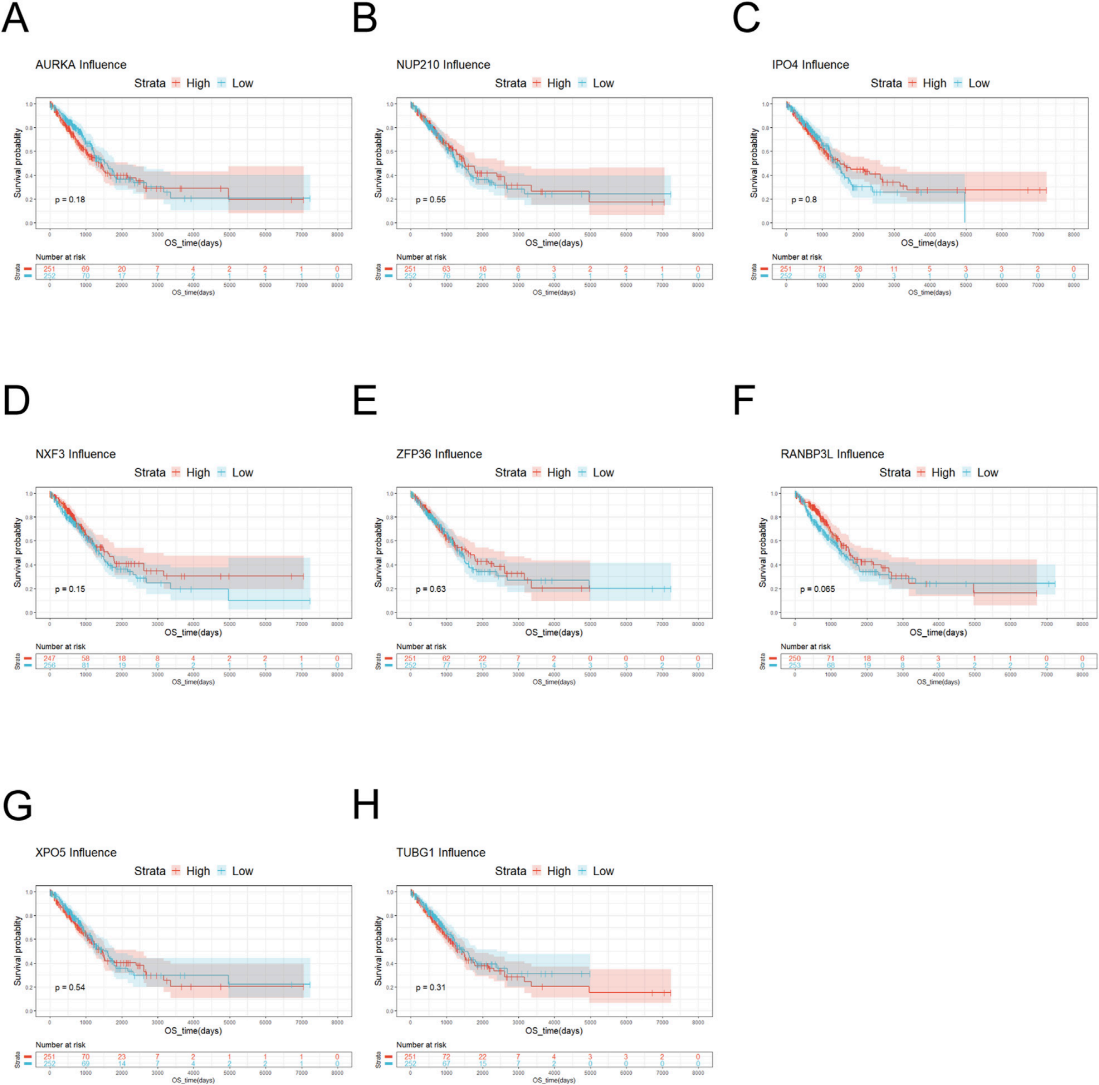
Survival analysis of key genes. **(A)** Survival analysis of AURKA showed that there was no significant difference in the survival probability between low-risk AURKA and high-risk AURKA. **(B)** The result of Survival analysis of NUP210 was similar to AURKA. **(C)** Survival analysis of IPO4 indicated that the survival probability of high-risk IPO4 was generally higher than that of low-risk IPO4. **(D)** Survival analysis of NXF3 manifested that the survival probability was higher for high-risk NXF3 than for low-risk NXF3. **(E)** Survival analysis was conducted on ZFP36. When the overall survival was less than 5,000 days, there was no significant difference in survival probability between low-risk and high-risk ZFP36. **(F)** Survival analysis of RANBP3L suggested that there was no significant difference in the survival probability between low-risk RANBP3L and high-risk RANBP3L. **(G)** Survival analysis of XPO5 exhibited that the survival probability was slightly higher for low-risk XPO5 than for high-risk XPO5. **(H)** Survival analysis of TUBG1 displayed that when the overall survival was less than 5,000 days, the overall survival of low-risk TUBG1 was higher than that of high-risk TUBG1.

The NUP family related-gene set was intersected with the differentially expressed genes to yield 19 related differentially expressed genes (Figure 2A). Survival analysis was performed for these differentially expressed genes. The survival analysis results of NUP155, NDC1, KPNA2, MAD2L1, NUP62CL, and POM121L2 were satisfactory (Figures 2B, C, 3, 4). Since NUP62CL had been shown to be valuable as a prognostic indicator of LUAD (Ren et al., 2021), this gene was excluded. It had been found that the interaction of NUP53 with NDC1 and NUP155 was indispensable during the assembly of the nuclear pore complex (Eisenhardt et al., 2014). Accordingly, only NUP155 and NDC1 were met our expectation.

Subsequently, we executed a survival analysis for NUP155 and NDC1 to find that low expression of NDC1 had a higher probability of survival than high expression, and the result for NUP155 was the same (Figures 2B, C). Next, we performed Western blot experiments to look at the relevant protein changes in the A549 and H1299 cell lines after inhibition of NUP155. The outcome indicated that the protein expression of NUP155 and NDC1 was significantly reduced after NUP155 knockdown (Figures 2D, E), while the protein expression of NUP155 and NDC1 was enhanced after overexpression of NUP155 (Figures 2F, G). After inhibition of NDC1, the protein expression of NDC1 in cancer cells was remarkably reduced, and the protein expression of NUP155 remained unchanged (Figures 2H, I). These results suggested that NUP155 played a role in NSCLC cells and that NDC1 was a downstream gene of NUP155.

### 3.3 NUP155 notably affected the function of NSCLC

**FIGURE 5:**
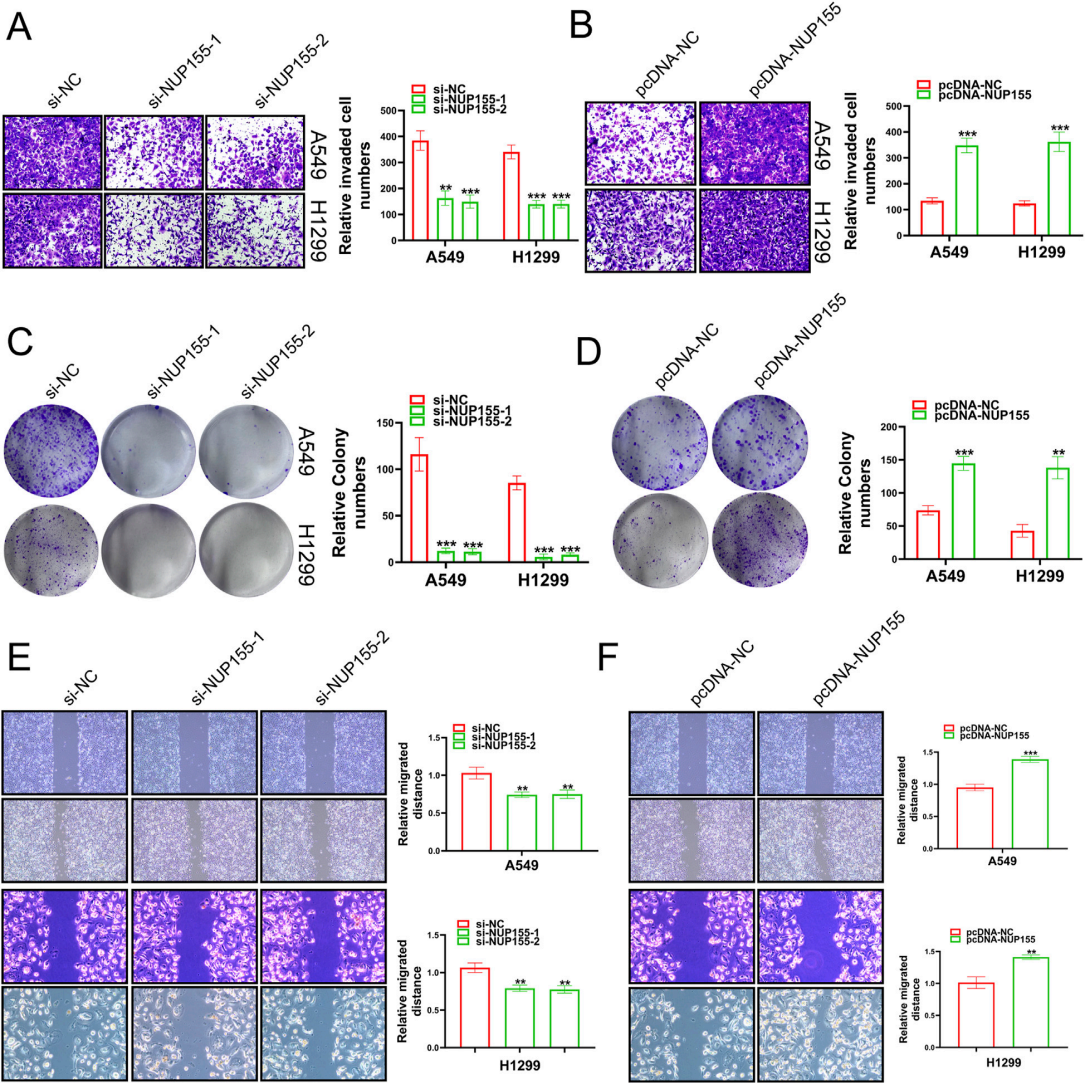
A series of experiments were conducted to detect the growth of cancer cells following changes in NUP155 expression. **(A)** After knockdown of NUP155, the results of Transwell assay showed that the invasion ability of cancer cells was significantly reduced. **(B)** After overexpression of NUP155, the results of Transwell assay showed that the invasion ability of cancer cells had enhanced. **(C)** After NUP155 knockdown, the proliferation ability of cancer cells was significantly decreased by clone formation experiment. **(D)** After overexpression of NUP155, the results of clone formation experiment displayed that the proliferation ability of cancer cells was increased. **(E)** After knockdown of NUP155, the results of scratch wound healing assay suggested that cancer cell migration ability was reduced. **(F)** After overexpression of NUP155, the results of scratch wound healing assay demonstrated that cancer cells had enhanced migration ability.

In an effort to unravel the mystery of NUP155 in NSCLC, we performed Transwell assays, clone formation, and scratch wound healing assay to detect the behavior of NSCLC cells following NUP155 changed. Inhibition of NUP155 significantly reduced the invasion, proliferation, and migration ability of NSCLC cells (Figures 5A, C, E). Overexpression of NUP155 enhanced cell invasion, proliferation, and migration (Figures 5B, D, F). The above experimental results suggested that NUP155 promoted the development of cancer cells.

### 3.4 NDC1 promoted cancer cell development

**FIGURE 6:**
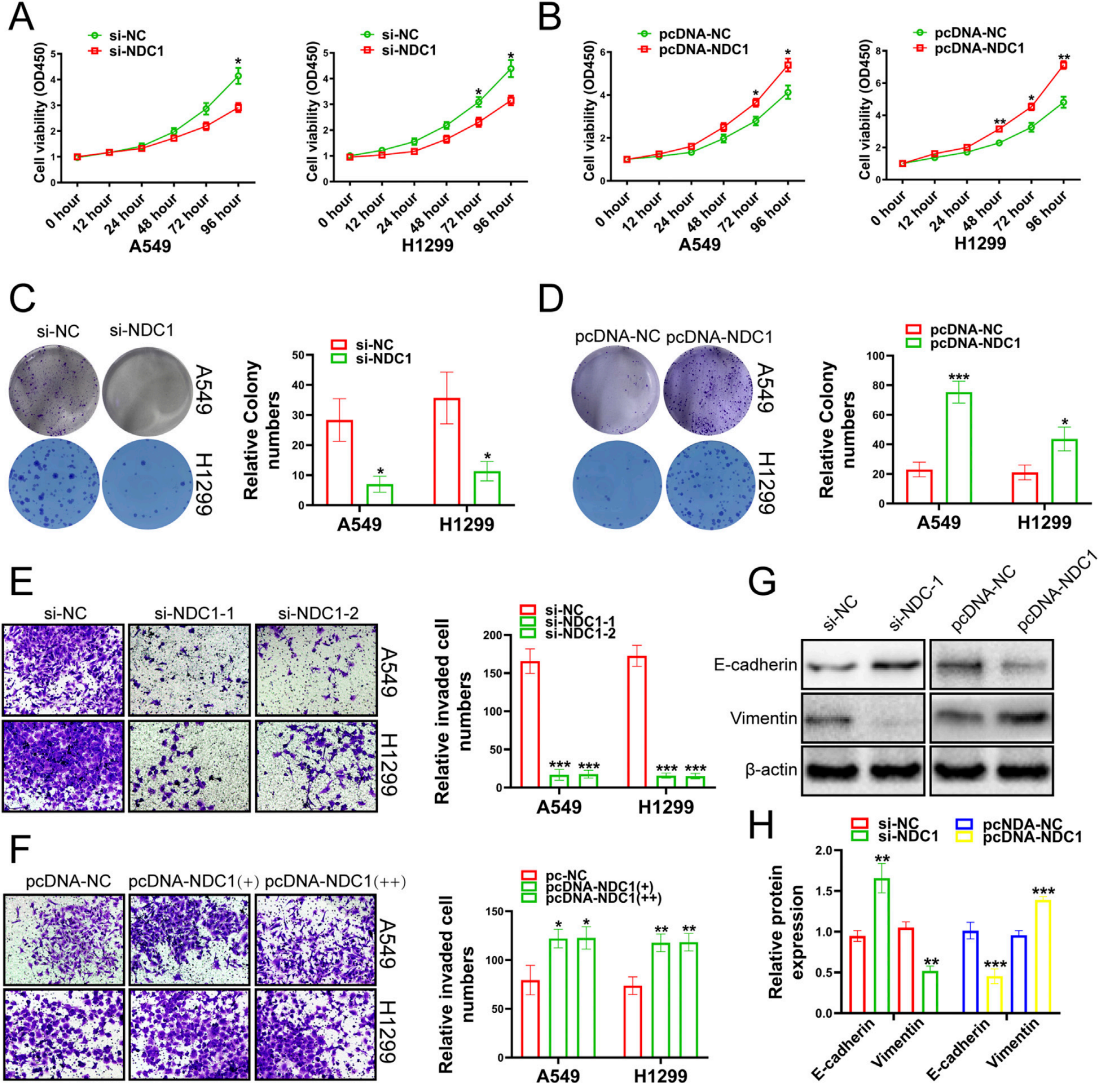
Growth changes of cancer cells were detected by experiments after the NDC1 expression changing. **(A)** The results of CCK-8 assay indicated that the viability of cancer cells was weakened after inhibition of NDC1. **(B)** After overexpression of NDC1, the results of CCK-8 assay showed that cancer cell viability was enhanced. **(C)** After NDC1 knockdown, the results of clone formation experiments showed that the migration ability of cancer cells had reduced. **(D)** After overexpression of NDC1, the results of Clone formation experiment confirmed that the migration ability of cancer cells was improved. **(E)** After NDC1 knockdown, the results of transwell assay showed that the invasion ability of cancer cells was weakened. **(F)** After overexpression of NDC1, the results of transwell assay confirmed that the invasion ability of cancer cells had enhanced. **(G)** After inhibition of NDC1, the expression of E-cadherin protein in cancer cells was enhanced, and the protein expression of Vimentin was weakened. After overexpression of NDC1, the expression of E-cadherin protein in cancer cells was decreased, and the protein expression of Vimentin was increased. **(H)** Histogram was used to show the results of E-cadherin and Vimentin in A549 cells.

Next, we investigated whether NDC1 exerted its function in lung cancer. The results of CCK-8 experiments showed that the cell viability, proliferation and migration ability of A549 and H1299 cell lines were meaningfully reduced by knockdown NDC1 (Figures 6A, C, E). Overexpression of NDC1 significantly increased cell viability, proliferation, and migration ability (Figures 6B, D, F). E-cadherin played an important role in tissue formation, tumor suppression, and Vimentin was closely associated with increased tumor cell invasiveness and metastasis (Goyal et al., 2021; van Roy and Berx, 2008), which both were the relevant marker of Epithelial-mesenchymal transition (EMT) (Busuioc et al., 2022). We respectively knocked down and overexpressed NDC1, and detected the protein expression fluctuations of E-cadherin and Vimentin. The results uncovered that the protein expression of E-cadherin was enhanced after NDC1 knockdown, while the protein expression of Vimentin was reversed. Overexpression of NDC1 attenuated the protein expression of E-cadherin and enhanced the protein expression of Vimentin (Figures 6G, H). The results demonstrated that NDC1 had a promoting effect on NSCLC cells. Compared with other studies, this study preliminarily identified the interaction between NUP155 and NDC1 and promoted the malignant progression of NSCLC.

## 4 Discussion

Lung cancer is one of the common causes of cancer-related deaths, and it has become the number one killer of human health. In China, approximately 631,000 people died from lung cancer each year, and the incidence of this cancer was still increasing (Wu et al., 2021). In the United States, there were approximately 247,270 new cases of lung cancer in 2020 (Alexander et al., 2020). It was reported that less than 7% of lung cancer patients survived within 10 years of diagnosis (Cheung and Juan, 2017). Lung cancer was divided into non-small cell lung cancer (NSCLC) and small cell lung cancer (SCLC), with NSCLC accounting for up to 85% (Molina et al., 2008). The main treatments for NSCLC were chemotherapy, radiotherapy and surgical modalities, but these were less effective, particularly in advanced cancer (Xu et al., 2024; Dong et al., 2021; Zhang et al., 2022). Fortunately, new treatments, such as targeted therapies for NSCLC, had improved patient’s prognosis over the last two decades (Wang et al., 2021). Our study found that there was an upstream and downstream regulatory relationship between NUP155 and NDC1, which were key nucleoporin proteins in the nuclear pore complex, and inhibiting the expression of NUP155 and NDC1 could inhibit the malignant progression of NSCLC cells. Our article also revealed that NUP155 and NDC1 were promising targets for the treatment of NSCLC.

It had been reported in the literature that NPCs were composed of nucleoporins (nups) and were associated with a variety of diseases. For example, genetic variants of Nup205 and Nup188 were found in the patients with congenital heart disease and situs inversus totalis or heterotaxy (Chen et al., 2023). Nup family genes such as NUP93, NUP188, and NUP205 had been found to play an important role in cilia-related cardiac left-right (LR) patterning (Del Viso et al., 2016; Marquez et al., 2021). This manifestation of the NUP family in the disease caught our attention, and we tried to study the relationship between the NUP family and NSCLC. In the previous study (Forest et al., 2022; Liu et al., 2023), bioinformatics analysis showed that NUP155 and NDC1 were highly expressed in NSCLC. However, *in vitro* cell models to verify the role of NUP155 and NDC1 in NSCLC were lacking, and the interaction between NUP155 and NDC1 was not well understood. Compared with these studies, this study preliminarily identified the interaction between NUP155 and NDC1 in an *in vitro* cell model, while promoting the malignant progression of NSCLC.

The NSCLC dataset was downloaded from the TCGA database, and 4,144 differentially expressed genes were obtained, including 2,322 high-expression genes and 1,822 low-expression genes. The gene set composed of these 4,144 genes intersected with the NUP family to obtain 21 related genes. After survival analysis, it was found that the analysis results of six genes, including NUP155, NDC1, KPNA2, MAD2L1, NUP62CL and POM121L2, were satisfactory. Of these, only NUP155 and NDC1 met our requirements. In the course of reviewing the literature, we found NUP155 work in a variety of cancers. High expression of NUP155 was identified in a hepatocellular carcinoma subgroup (Boyault et al., 2007). In breast invasive carcinoma (BRCA) cells, NUP155 was detected to be upregulated, and it played a tumorigenic role in BRCA (Wang et al., 2024). Expression of the p53 status, Nup155 and FTSJ1 expression was found to be associated with mouse and human liver cancer (Holzer et al., 2019). While NDC1 was found to be overexpressed in the NSCLC cell lines H1299 and A549, researchers believed that inhibition of NDC1 expression might be a therapeutic modality for NSCLC (Qiao et al., 2016). Therefore, we studied and analyzed the performance of these two genes in NSCLC. The results of survival analysis indicated that both were low-risk genes with a higher probability of survival. After knockdown of NUP155, the protein expression of A549 and H1299 in cancer cells decreased, and the invasion, proliferation and migration ability of cancer cells were also weakened. Overexpression of NUP155 manifested the opposite in cancer cells. Similar experiments were performed on NDC1, and the overall performance of NDC1 was similar to that of NUP155. The results of Western blot suggested that NDC1 increased with the enhancement of NUP155, and NDC1 was the downstream gene of NUP155. We considered that NUP155 might promote the rate of nuclear division by attracting NDC1 to upregulate the formation of nuclear pore complexes, thereby increasing the replication rate and function of NSCLC cells, thereby promoting the malignant progression of NSCLC.

Collectively, we obtained the result from the above studies that NUP155 advanced the progression of NSCLC cells by promoting NDC1 expression. However, there were some limitations in this study. Firstly, the role of NUP155 and NDC1 were investigated in only two cell lines and the lack of *in vivo* validation. Secondly, the interaction between NUP155 and NDC1 were investigated in preliminary stage. The underlying mechanism needed further investigation. Thirdly, given the role of NUP155 or NDC1 in normal cellular processes, there might be off-target effects with drugs targeting NUP155 or NDC1. This needed to be confirmed and circumvented by follow-up research.

## 5 Conclusion

NUP155 and NDC1 could promote the malignant functions of NSCLC cells. Moreover, NUP155 was positively correlated with NDC1. In this study, we identified that NUP155 had a potential to be a targeted gene for NSCLC, and it is a promoter of NSCLC cells by regulating the expression of NDC1.

## Data Availability

The datasets presented in this study can be found in online repositories. The names of the repository/repositories and accession number(s) can be found in the article/supplementary material.
